# Genome editing of *Caenorhabditis briggsae* using CRISPR/Cas9 co-conversion marker *dpy-10*

**DOI:** 10.17912/micropub.biology.000171

**Published:** 2019-10-11

**Authors:** Sarah M Cohen, Paul W Sternberg

**Affiliations:** 1 Division of Biology and Biological Engineering 156-29, California Institute of Technology, Pasadena, CA 91125, USA

**Figure 1:**
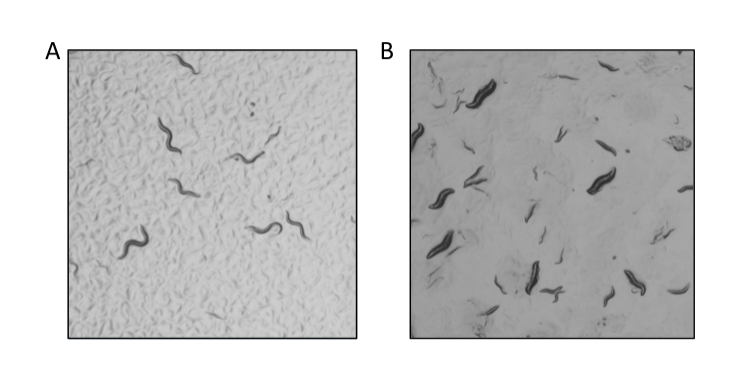
(A) Wildtype AF16 worms as compared with (B) *Cbr-dpy-10*, which shows the Dumpy phenotype.

## Description

Genomic editing of the roundworm *Caenorhabditis elegans* using the CRISPR/Cas9 system has allowed for widespread creation of null mutants – vital for scientific understanding of this model organism. A closely-related nematode species, *Caenorhabditis briggsae*, is emerging as an alternative model organism to better understand how findings in *C. elegans* can broaden and develop the larger field of nematology (Gupta *et al.* 2007). To that end, we have developed an effective and efficient co-conversion CRISPR/Cas9 system for use in *C. briggsae* using the gene *dpy-10*.

We modified the universal STOP-IN cassette method, as described by Wang *et al.* (2018), for use in *C. briggsae* (Wang *et al.* 2018). Using the wildtype AF16 strain, we tested the method by choosing the gene *Cbr-dpy-10* because of its readily observed predicted phenotype. *Cbr-dpy-10* is a predicted one-to-one ortholog of *C. elegans* *dpy-10*, which encodes a protein important for cuticle development and has a phenotype characterized by short, fat animals relative to wild type (Brenner 1974).

A universal STOP-IN allele of *Cbr-dpy-10, sy1387,* was generated and confirmed by genotype sequencing. The expected phenotype was subsequently observed, consistent with the creation of a null allele (Figure 1). Surprisingly, *sy1387* is dominant. Injections of 20 animals produced 15 successful injections; 247 animals from these 15 injections (F1) were singled out and allowed to self-fertilize to produce F2. 81% of the F1 Dumpy progeny were homozygous as evidenced by their segregation of only Dumpy progeny – one of these candidates became *sy1387*. 2% of the F1s did not produce progeny. 17% of the 247 F1 Dumpy progeny were heterozygotes and had a mixture of Dumpy and non-Dumpy progeny; these non-Dumpy F2s only produced non-Dumpy progeny. This discovery of a dominant mutation will allow for more effective use of this as a co-conversion marker when screening for other mutations. We used *Cbr-dpy-10* as a potential co-CRISPR marker for a second target that will be described elsewhere. We used PCR to detect insertion of a STOP-IN cassette at this other locus; we screened 39 Dumpy strains to obtain 11 candidates for our target gene, from which we have three STOP-IN alleles.

## Methods

We used the universal STOP-IN cassette method essentially as described in Wang *et al.* (2018). Potential guide sequences were followed by a 5’-NGG-3’ PAM site and were close to the start codon ATG of the target gene. The guide sequence for *Cbr-dpy-10* used in this protocol was ATTCGCGTCAGATGATGTAC, located at the beginning of the gene’s second exon. To detect the stop-in insertion into *Cbr-dpy-10*, we used forward primer GAAAAACAACGGCAGAGACG and reverse primer TCCGCTTCCATAAGCACCAC.

In *sy1387*, the second exon of *Cbr-dpy-10* (shown below) was changed with the 41 basepair insert highlighted in red. This caused the early introduction of a stop codon (underlined) and a subsequent frameshift.

CTATCGAACATTCTCGCTGACAACGAACTATTCGCGTCAGATGATGGAAGTTTGTCCAGAGCAGAGGTGACT
AAGTGATAAGCTAGCTACCGGTGTGTCACAGGTCTTCAAATCGGATTTAGTTTGTTCTCATTCGTTATCGTT
TGTGCCCTTCCTATTATGTACAATCATGTCCAGAACACAATTACTTATGTTGACAGAGAAATAGTGGCTTAT
TGCGAA

## Reagents

All CRISPR/Cas9 system reagents were ordered from IDT except for the Cas9 protein, which was kindly provided by Tsui-Fen Chou. Sequences were downloaded from WormBase.

Strain Generated:

PS8520 *Cbr-dpy-10* (*sy1387*) II
